# Biased visibility in Hi-C datasets marks dynamically regulated condensed and decondensed chromatin states genome-wide

**DOI:** 10.1186/s12864-020-6580-6

**Published:** 2020-02-22

**Authors:** Keerthivasan Raanin Chandradoss, Prashanth Kumar Guthikonda, Srinivas Kethavath, Monika Dass, Harpreet Singh, Rakhee Nayak, Sreenivasulu Kurukuti, Kuljeet Singh Sandhu

**Affiliations:** 1 Department of Biological Sciences, Indian Institute of Science Education and Research (IISER) – Mohali, Knowledge City, Sector 81, SAS Nagar, 140306 India; 2 Department of Animal Biology, School of Life Sciences, University of Hyderabad (UoH), Central University, Prof. CN Rao Road, P O, Gachibowli, Hyderabad, Telangana 500046 India

**Keywords:** Hi-C, 3D genome, Chromatin condensation, Lamina associated domains, CTCF

## Abstract

**Background:**

Proximity ligation based techniques, like Hi-C, involve restriction digestion followed by ligation of formaldehyde cross-linked chromatin. Distinct chromatin states can impact the restriction digestion, and hence the visibility in the contact maps, of engaged loci. Yet, the extent and the potential impact of digestion bias remain obscure and under-appreciated in the literature.

**Results:**

Through analysis of 45 Hi-C datasets, lamina-associated domains (LADs), inactive X-chromosome in mammals, and polytene bands in fly, we first established that the DNA in condensed chromatin had lesser accessibility to restriction endonucleases used in Hi-C as compared to that in decondensed chromatin. The observed bias was independent of known systematic biases, was not appropriately corrected by existing computational methods, and needed an additional optimization step. We then repurposed this bias to identify novel condensed domains outside LADs, which were bordered by insulators and were dynamically associated with the polycomb mediated epigenetic and transcriptional states during development.

**Conclusions:**

Our observations suggest that the corrected one-dimensional read counts of existing Hi-C datasets can be reliably repurposed to study the gene-regulatory dynamics associated with chromatin condensation and decondensation, and that the existing Hi-C datasets should be interpreted with cautions.

## Background

The three-dimensional genome organization is tightly linked with the regulation of essential genomic functions like transcription, replication and genome integrity [1–5]. While the significance of genome organization has been realized for decades, the comprehensive evidence emerged somewhat recently through the advent of proximity ligation based techniques like Chromosome Conformation Capture (3C), Circular-3C (4C), 3C-Carbon-Copy (5C) and High-throughput 3C (Hi-C) [6–10]. It is recognized that the eukaryotic genome is hierarchically organized into self-interacting topologically associated domains (TADs), which can have distinct chromatin states that are insulated from neighbourhood through boundaries marked with CCCTC-binding factor (CTCF), Cohesins, ZNF143 and TOP2b factors [11–14]. The TADs are ancient genomic features and are depleted in evolutionary breakpoints inside [15, 16]. It is proposed that chromatin extrudes through the ring formed by the Cohesins until the chromatin encounters the CTCF insulator, a model known as ‘loop extrusion’ model [17–20]. CTCF binding is transiently lost during pro-metaphase, which coincides with the loss of TAD structures during M-phase [21–23]. Systematic depletion of CTCF and Cohesins also leads to de-insulation and partial disruption of TADs [24, 25]. An array of studies has shown that TADs function as basic units of three-dimensional (3D) genome organization and dynamically associate with the epigenetic states of genes, including replication timing, during development and differentiation [26–34]. How these dynamical epigenetic states of TADs are regulated is not entirely clear. One of the ways this can be achieved is through chromatin condensation and decondensation, implying inactive and active states of TADs respectively [2, 35–38] (Benabdallah et al. 2018, bioRxiv). While it is established that the gene-poor and transcriptionally inactive domains locate towards nuclear periphery and mostly remain stably condensed, with exceptions of local gene-specific alterations during differentiation [39, 40], the dynamics of chromatin condensation and decondensation in the other regions of the genome largely remains under-explored. Condensation and decondensation of chromatin is generally studied through microscopic methods. In this study, we demonstrate that the condensed and decondensed states of chromatin domains can be directly inferred from the one-dimensional Hi-C read counts.

Yaffe and Tanay have shown that Hi-C datasets have systematic bias due to differential ligation efficiencies of restriction fragments of different lengths, differential amplifications of fragments of different GC contents, and differential mappability of sequences [41]. Several methods have since been developed to normalize the aforementioned systematic biases. These methods can be broadly categorized into two classes, the ones that define the aforementioned biases explicitly in the algorithm and the ones that do not define the source of bias and instead adopt an implicit approach based on fractal folding of the chromatin and the equal visibility of all genomic loci [41–45]. In this study, we show that the differential visibility of genomic loci to the restriction endonucleases used in Hi-C protocols induct potential bias in Hi-C data. Hi-C reads are significantly depleted for the interactions impinging from condensed heterochromatin domains, and this bias is not appropriately corrected by existing computational methods. By repurposing the observed bias, we first demonstrate that the bias in one-dimensional read counts of Hi-C datasets reliably marks the known condensed and decondensed domains in the genome and then highlight the developmentally regulated dynamics of condensed and decondensed states of chromatin genome-wide.

## Results

### Biased visibility in Hi-C data marks condensed and decondensed chromatin domains

**Fig. 1:**
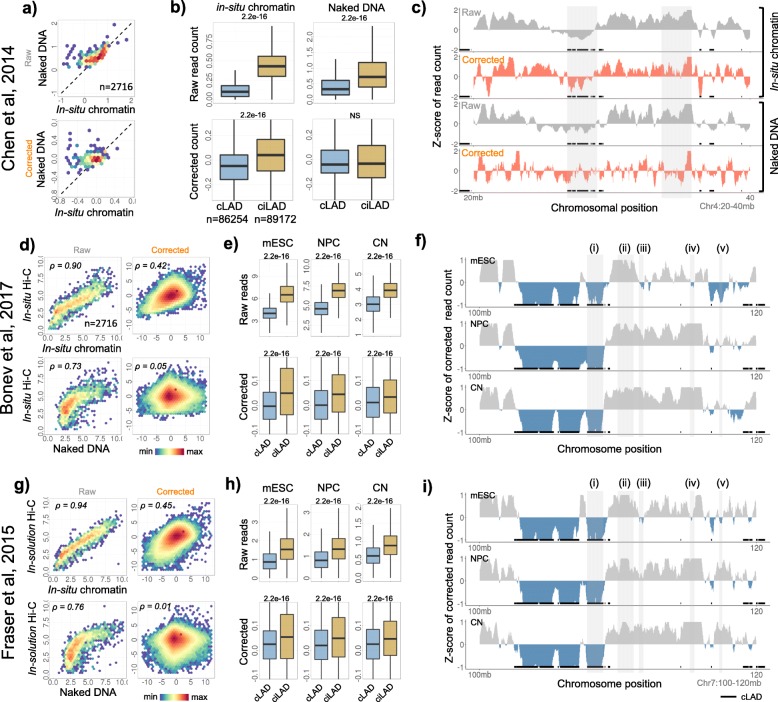
Biased visibility of chromatin domains in in-situ Hi-C datasets. **a** Scatter plots of raw and corrected read counts (per Mb) in in-situ digested chromatin vs. *in-solution* digested naked DNA. **b** Distribution of raw and corrected read counts of in-situ digested chromatin and in-solution digested naked DNA in cLADs and ciLADs. *P*-values were calculated using one-tailed Mann-Whitney U tests. **c** Illustrative example of raw and corrected read counts of in-situ digested chromatin and *in-solution* digested naked DNA along chr4: 20-40 Mb region. **d** Top: scatter plots of raw and corrected read counts in in-situ Hi-C and in-situ digested chromatin. Bottom: scatter plots of raw and corrected read counts in in-situ Hi-C and *in-solution* digested naked DNA. *‘ρ’* represents the Spearman’s correlation coefficient. **e** Distribution of raw and corrected read counts of in-situ Hi-C datasets in cLADs and ciLADs. *P*-values were calculated using two-tailed Mann-Whitney U test. **f** Illustrative examples of corrected read counts of in-situ Hi-C datasets along chr7: 100–120 Mb. Regions (i) and (ii) mark constitutively condensed and decondensed regions respectively. Regions (iii)-(v) mark cell-type specific condensed and decondensed states. (ii) (**g-i**) Same as d-f, but for *in-solution* Hi-C data obtained from Fraser et al.

Restriction endonucleases are the preferred choice of chromatin digestion in Hi-C studies. We first tested if the in-situ restriction digestion of chromatin is uniform in the genome. This could be tested by comparing the sequencing data of restriction endonuclease digested chromatin and the naked DNA. Regional depletions in read counts obtained from digested chromatin when compared with the reads obtained from digested naked DNA would mark the biased restriction digestion of chromatin. Towards this, we obtained the ‘Restriction Endonuclease Digestion coupled with sequencing’ (RED-seq) data of in-situ restriction digested chromatin and *in-solution* restriction digested naked DNA of mouse embryonic stem cells (mESC) from Chen et al. [46]. We calculated the read counts for 10 kb bins of the mouse genome and normalized by the total reads. We further corrected the read counts for restriction site density (RE-density) and the GC content of the bins using loess regressions, in that order (Methods, Fig. S1a-b). The scatter-plot of restriction digested naked DNA and in-situ digested chromatin showed skew towards naked DNA axis marking the inefficient digestion of certain genomic regions in chromatin but not in naked DNA (Fig. 1a). This suggests that chromatin structure influences its own digestibility. The likely explanation is that the decondensed chromatin is readily digested while heterochromatin domains have limited accessibility to restriction endonuclease due to compact packing.

To further assess the above hypothesis, we obtained the Lamina Associated Domains (LADs), which are known heterochromatin domains attached to the nuclear periphery in condensed form [35, 47]. We calculated the raw and corrected one-dimensional (1D) read counts in the constitutive LADs (cLADs) and constitutive inter-LADs (ciLADs) in mESC. As shown in the Fig. 1b-c, cLADs exhibited significantly less raw read counts as compared to ciLADs in in-situ digested chromatin as well as in *in-solution* digested naked DNA, suggesting that the reads from digested naked DNA had bias likely due to varying densities of restriction sites and distinct GC compositions of cLADs and ciLADs (Fig. 1b-c, *p* < 2.2e-16). The read counts corrected for RE-density and GC content, however, exhibited bias only in the in-situ digested chromatin and not in the naked DNA, highlighting that the cLADs were relatively inaccessible to restriction endonuclease likely due to condensed nature of the chromatin (Fig. 1b-c, *p* < 2.2e-16). We further identified the chromatin domains significantly enriched (decondensed) or depleted (condensed) in corrected read counts (Methods, Fig. S1). Overall, 77% of the length covered by condensed domains were within cLADs and 23% mapped to ciLAD regions, marking the novel condensed domains other than nuclear lamina associated domains (Fig. S1d).

These analyses suggest that the molecular techniques, like Hi-C, involving restriction digestion as preferred method of chromatin fragmentation might suffer from bias in final readouts. We, therefore, expanded our analyses to 45 Hi-C datasets (21 in-situ Hi-C, 11 *in-solution* Hi-C and 8 single cell Hi-C, 2 Drosophila Hi-C, 2 DNase Hi-C, 1 native Hi-C) and obtained the processed reads (Fig. 1d-f, Fig. S2, Table S1, Methods). One-dimensional read counts were corrected for the density of restriction sites and GC content as earlier. Through analysis of mESC data, we observed that the read counts from in-situ Hi-C had a significant correlation with the read counts obtained from in-situ digested chromatin, but exhibited skewed scaling towards the read-counts of digested naked DNA (Fig. 1d). This suggested that the in-situ Hi-C reads exhibited bias similar to the one observed in in-situ digested chromatin. As shown in the Fig. 1e-f and Fig. S2, the corrected read counts exhibited enrichment in ciLADs and depletion in cLADs (*p* < 2.2e-16). Again, 70% of the total length covered by the condensed domains was within cLADs and 30% was within ciLAD regions, marking the condensed domains other than LADs. (Fig. S1d). Our observations with cLADs and ciLADs were consistent with different in-situ Hi-C datasets, including single-cell Hi-C, generated using distinct restriction endonucleases (Fig. 1d-i and S2, *p* < 2.2e-16). We illustrated the examples of condensed domains that mapped to cLADs, to ciLADs and the ones that exhibited cell-type specificity in the Fig. 1f and S2.

We also showed that the observed differences for the cLAD and ciLADs were not due to processing of Hi-C sequencing data through Hi-C User Pipeline (HiCUP). We observed the bias in reads simply processed through bowtie too (Fig. S3a, *p* < 2.2e-16, Methods). Further, the biased visibility was not the property of in-situ Hi-C only, but was also observed in *in-solution* Hi-C (Fig. 1g-i, S3b, *p* < 2.2e-16). As shown in the Fig. S3c, the corrected reads from in-situ Hi-C exhibited good correlation with those from *in-solution* Hi-C in the same cell-type (mouse fetal liver) from the same study [48]. These analyses suggest that the visibility bias is not affected by the method of ligation and that the source of bias is likely the difference in accessibility to the restriction endonucleases, and not the difference in ligation.

**Fig. 2:**
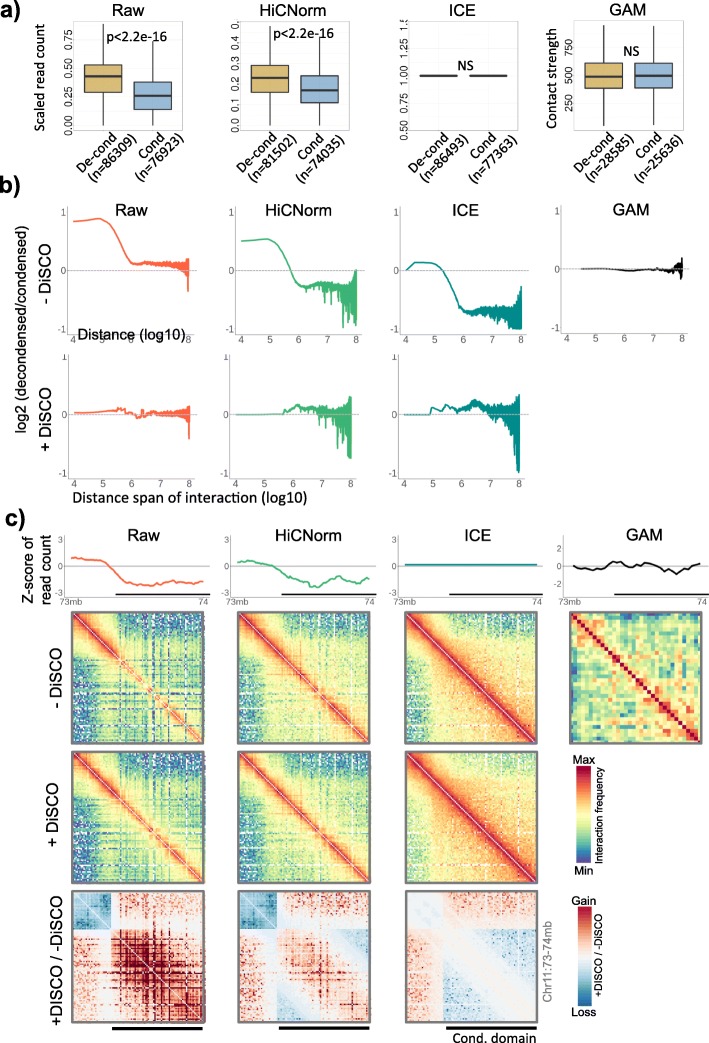
Bias in explicitly and implicitly normalized Hi-C, and GAM datasets. **a** Distribution of 1D read-counts of decondensed and condensed domains in raw, HiCNorm-corrected, ICE-corrected Hi-C and GAM datasets of mESCs. Values were scaled from 0 to 1. P-values were calculated using two-tailed Mann-Whitney U tests. **b** Upper panel: ratio of interaction frequencies of decondensed-to-decondensed and condensed-to-condensed interactions as a function of genomic distance in raw, HiCNorm-corrected, ICE-corrected and GAM datasets. Lower panel: plots after DiSCO correction. **c** Illustrative examples of raw, HiCNorm-corrected, and ICE-corrected data before and after DiSCO correction. Ratio matrices in the bottom panel show gain and loss of signals after DiSCO correction. GAM data is shown on extreme right for comparison. Additional examples are given in the Fig. S4

HiCNorm, an explicit method of Hi-C correction, failed to remove the bias in the read counts, supporting that the observed bias was independent of known systematic biases of Hi-C data (Fig. 2a & S4, *p* < 2.2e-16). Iterative correction, an implicit method, normalized the read counts attributing to its intrinsic nature of polishing the Hi-C matrices for equal visibility of all loci without defining the bias at first place (Fig. 2a & S4). Data obtained from Genome Architecture Mapping (GAM) [49], which directly obtains the co-localized DNA segments through large number of thin nuclear sections and does not involve any restriction digestion and ligation steps, did not exhibit any bias in the read counts. By comparing GAM and ICE-corrected Hi-C data, we further observed that ICE merely lifted the background and the obscure signals in the contact matrices. In the process of lifting the obscure signals in the poorly digested condensed regions, ICE inadvertently lifted the long-range background interactions among condensed domains as shown in the Fig. 2b and S4. To address this, we proposed that the ICE-corrected Hi-C datasets needed a further distance dependent optimization of interaction frequencies. We termed this additional step as Distance Sorted Contact Optimization (DiSCO) and implemented it on raw, HiCNorm-corrected and ICE-corrected Hi-C matrices. As shown in in the Fig. 2b, the method corrected the distance dependent bias in interaction frequencies of condensed and decondensed domains. Though DiSCO corrected only the distance dependent bias when implemented on the raw data, it was able to balance the contact matrices for most of the biases when combined with the ICE. In particular, the long-range interactions of condensed domains, which were inadvertently lifted by ICE, were corrected by DiSCO, and the short-range interactions remained largely unaltered (Fig. 2b-c & S4). Inclusion of DiSCO did not reintroduce the coverage bias in the ICE-corrected 1D read counts, suggesting the overall suitability of the approach (Fig. S4b). The comparison with the GAM matrices also showed sub-TAD structures and other types of interactions in the condensed domains (Fig. 2c & S4c-e), which were clearly not captured by raw or any of the corrected Hi-C matrices, suggesting the inherent limitation of Hi-C in resolving the organization of condensed chromatin.

**Fig. 3:**
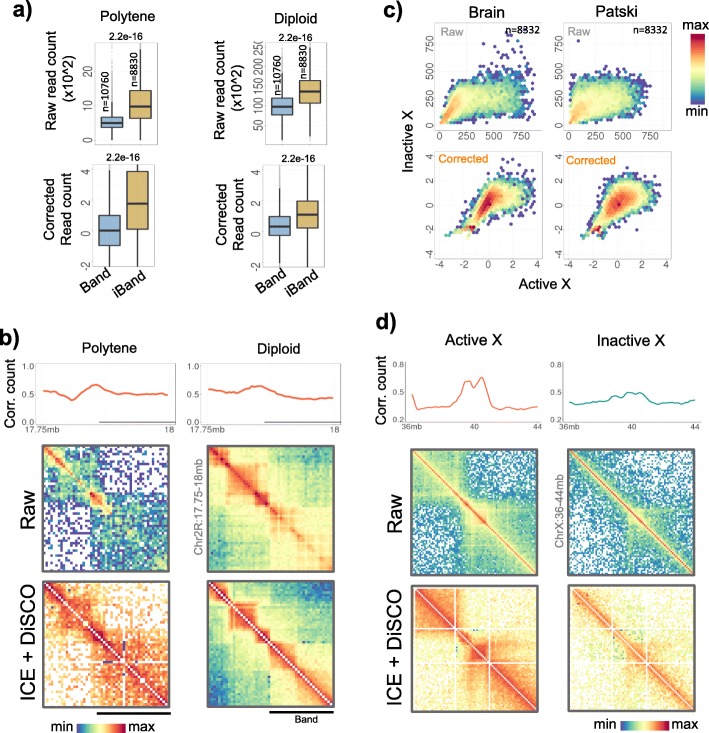
Low visibility of polytene bands and inactive X-chromosome. **a** Distribution of raw and corrected read counts in band and inter-band regions of polytene chromosome and the corresponding regions in diploid chromosome. P-values were calculated using two-tailed Mann-Whitney U tests. **b** Illustrative examples of read counts and contact maps in band and inter-band regions (chr2R: 17.5-18 Mb) of polytene and diploid chromosome. Band regions are marked as horizontal line below the line plots. **c** Scatter plots of raw and corrected DNase-Hi-C read counts of active vs. inactive x-chromosomes in Brain and Patski cells. **d** Illustrative examples of corrected read counts and contact maps of chrX: 36–44 Mb region in active and inactive X-chromosome

To further scrutinize the differential digestion of condensed and decondensed domains, we obtained the Hi-C data of Drosophila polytene chromosome, which is a typical example of spatially condensed (polytene bands) and decondensed (inter-bands) domains [50]. The Hi-C reads were mapped and corrected as earlier. The analysis suggested that the polytene bands had lesser enrichment of corrected reads as compared to inter-band regions on both the polytene chromosome and the normal diploid chromosome (Fig. 3a, *p* < 2.2e-16). We illustrated our observations through examples in the Fig. 3b. On similar lines, we analysed the DNase Hi-C data for active and inactive X-chromosomes in brain and patski cells [51]. As shown through the scatter plots in Fig. 3c and examples in Fig. 3d, the X-chromosome had regions that were more visible in active X-chromosome and less visible in inactive X-chromosome. This suggested that the bias due to differential chromatin accessibility existed in both restriction endonuclease digested and DNase digested Hi-C datasets.

These observations highlight that: 1) the observed bias in corrected 1D Hi-C read counts is independent of known systematic biases of Hi-C; 2) the bias captures the condensed and decondensed states of chromatin domains reliably, and 3) the existing computational approaches of Hi-C normalization need further optimization for the condensed and decondensed domains.

### Dynamics of condensed and decondensed domains

**Fig. 4:**
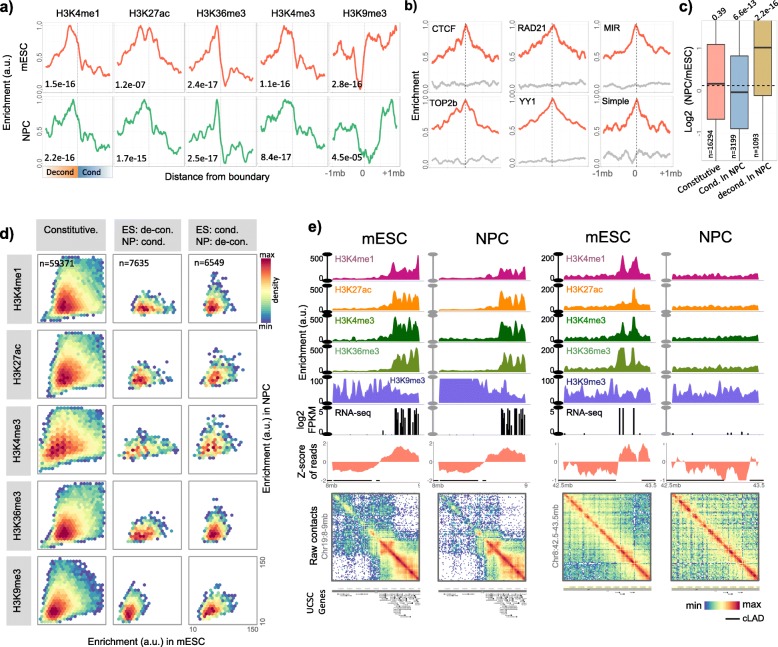
Developmental dynamics of chromatin condensation and decondensation. **a** Aggregation plots of histone modifications +/− 1 Mb around the boundary of decondensed and condensed domains in mESC and NPC. P-values were calculated using two-tailed Mann-Whitney U tests by comparing mean enrichment values in the bins of condensed and decondensed domains. **b** Enrichment of CTCF, RAD21, YY1, TOP2b binding, MIR and simple repeats +/− 1 Mb around domain boundaries (red) and around domain centres (grey). **c** Boxplots representing change in gene expression in the chromatin domains that were constitutively present in mESC and NPC, the ones that switched to condensed state in NPC from decondensed state in mESC and vice-versa. *P*-values were calculated using two-tailed Mann-Whitney U tests of RPKM values in mESC and NPC. **d** Scatter plots of histone modifications in domains that remained unchanged in mESC and NPC, and the ones that switched from decondensed to condensed or vice-versa in mESC and NPC. **e** Examples of decondensed and condensed domains that remained consistent in mESC and NPC (left), and a decondensed region in mESC that switched to condensed state in NPC (right).

To assess if the condensed and decondensed domains identified from restriction digestion bias in the ciLAD regions had functional significance, we analysed their dynamics during mouse embryonic stem cell (mESC) differentiation to neuronal progenitor cells (NPC) to cortical neurons (CN). As shown in the Fig. S5a, the differentiation from mESC to NPC exhibited greater overall change in corrected read counts as compared to NPC to CN differentiation. We, therefore, focussed on mESC to NPC differentiation to assess the developmental regulation of chromatin condensation and decondensation. We first mapped the histone modification and CTCF binding data around boundaries of domains by placing all decondensed domains upstream and all condensed domains downstream to the domain boundaries (Fig. 4a & S5b). We observed enrichment of active and inactive histone marks in decondensed and condensed domains respectively with transitions around boundaries that were marked with CTCF, RAD21, YY1, TOP2b, MIR and simple repeat elements (Fig. 4a-b & S5c, *p* = 4.5e-05 to 2.2e-16). Total 27.7% of condensed ciLAD domains in mESC were decondensed in NPC and 13.5% decondensed domains in mESC were condensed in NPC, suggesting significant cell-type specificity of domains identified through biased visibility in Hi-C data (Fig. S5d). Genes exhibiting condensation during differentiation switched to repressed state and the ones showing decondensation switched to active state (Fig. 4c, *p* = 6.6e-13 & 2.2e-16). Through scatter plots of histone marks between mESC and NPC cells, we observed that the condensation of open chromatin domains during differentiation was associated with the coherent change of active to inactive chromatin states (Fig. 4d). Similarly, the domains that exhibited decondensation during differentiation switched to active states from inactive chromatin states (Fig. 4d). Enrichment of neuronal development related terms among genes exhibiting decondensation, and the metabolism related terms among genes exhibiting condensation during ESC-to-NPC differentiation coherently supported the underlying functional significance (Fig. S6a). We illustrated a few examples of constitutive and cell-type specific chromatin domains in the Fig. 4e and S5e-g. These observations not only highlight the developmental regulation of chromatin domains identified in the study, but also argue strongly against the dismissal of restriction digestion bias merely as an artefact.

**Fig. 5:**
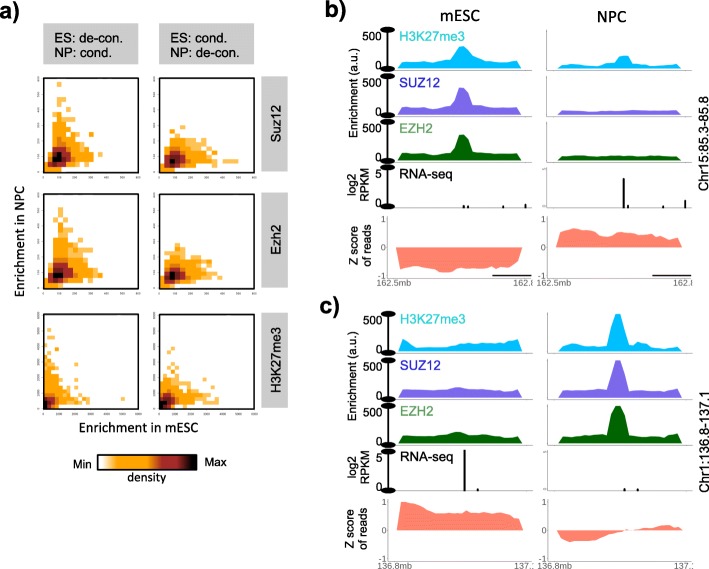
Polycomb association of non-LAD condensed domains identified through visibility bias. **a** Scatter plots of Suz12, Ezh2 and H3K27me3 in domains that switched from decondensed to condensed or vice-versa in mESC and NPC. **b-c** Examples of decondensed and condensed domains that switched their status in mESC and NPC

While the shift of active chromatin states towards the axis that represented the decondensed state of the involved domain in mESC or NPC was clear in Fig. 4d, the repressive chromatin state (H3K9me3 mark) showed relatively subtle shift only. This was coherent with the earlier reports that suggested only subtle changes in H3K9 tri-methylation profiles during mESC differentiation [40, 52]. We instead observed that the enrichment of polycomb associated marks and proteins (H3K27me3, Suz12 and Ezh2) exhibited shift towards the axis that represented condensed state of chromatin domains (Fig. 5). Polycomb association was also supported by the significant overlap of genes exhibiting decondesation during ESC-to-NPC transition with the Suz12 targets, Eed targets, PRC12 targets, and the targets of bivalent histone modifications (Fig. S6b, right panel). These results imply that the non-LAD condensed domains uncovered in this study are likely representative of polycomb-repressed chromatin.

**Fig. 6:**
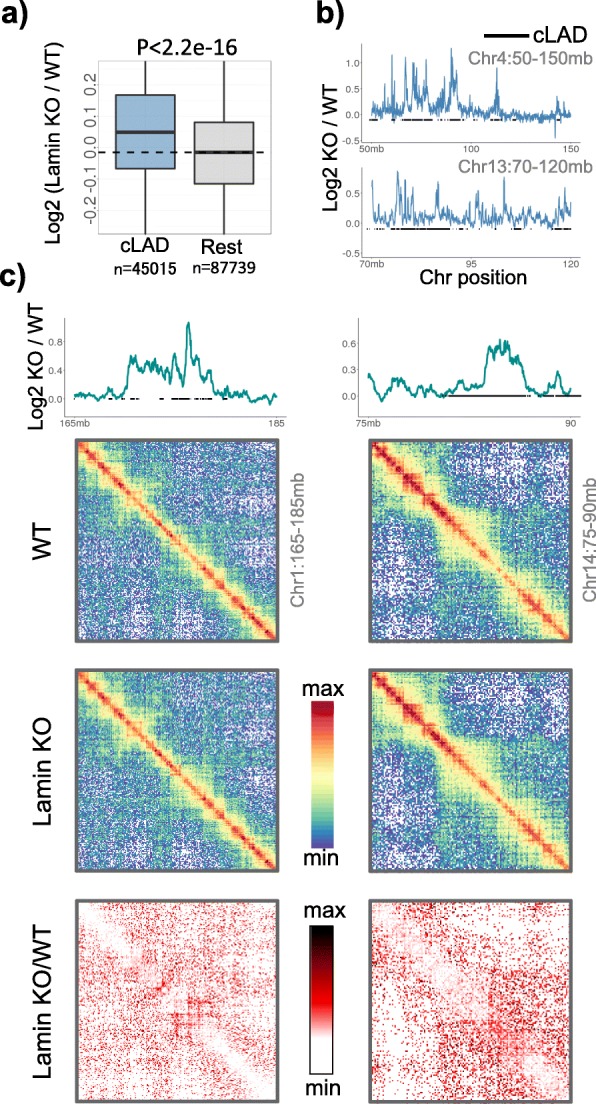
Capturing chromatin decondensation in Lamin KO cells. **a** Boxplots representing change in Hi-C read counts in the LAD domains after Lamin knock out in mESC cells. P-value was calculated using two-tailed Mann-Whitney U test. **b-c** Examples representing change in Hi-C read counts and contact matrices in WT and Lamin KO cells. The bottom panel of matrices represent the Lamin KO to WT fold-change in interaction frequencies

Chromatin condensation and decondensation can be induced by knocking out certain factors like Lamins. We, therefore, tested if such experimentally induced decondensation of LADs can be captured through analysis of 1D Hi-C reads of Lamin knock out (KO) cells. We obtained the Hi-C data for wild-type (WT) and Lamin (*Lmb1, Lmb2, Lmna*) KO mouse embryonic stem cells from Zheng et al. [53]. As shown in the Fig. 6a, cLADs exhibited significant increase in 1D read counts in Lamin KO over WT when compared to rest of the genome (*p* < 2.2e-16). We illustrated this observation through examples in Fig. 6b-c. Our observations highlight that the 1D Hi-C read-counts alone can capture the decondensation of LAD domains after lamin deletion.

## Discussion

To study the dynamics of gene regulatory activity in response to environmental and developmental clues, mapping chromatin accessibility remained an important task over decades. While DNase-I has been a preferred choice to digest the open chromatin due to relatively lesser sequence specificity of the enzyme, only few studies attempted restriction endonucleases to identify accessible regions in the chromatin [46, 54, 55]. On the contrary, restriction endonucleases have been extensively used to digest the chromatin, presumably in an unbiased manner, in proximity ligation based techniques like 3C, 4C, 5C and Hi-C [6–9]. In techniques like 3C, which has limited scope in terms of regions to be tested for spatial interactions, the efficiency of restriction digestion of the regions of interest can be tested and controlled [56]. For high throughput assays like Hi-C, data on genome-wide assessment of digestion efficiency is rarely seen in the research articles and the associated supplementary materials. Prior to prolonged restriction digestion, in-situ Hi-C involves treatment of nuclei with 0.1–0.5% sodium dodecyl sulphate (SDS) at 62 °C for 5–10 min, followed by quenching of SDS using 10% Triton X-100 at 37 °C for 15 min [8, 45, 57]. The heat and the detergent treatments are expected to open the chromatin and ease the accessibility of DNA to restriction endonuclease. We have shown that despite these treatments, the visibility of condensed chromatin to restriction endonucleases is significantly limited. As a result, interactions within heterochromatin and the ones impinging onto heterochromatin are under-represented in the current Hi-C datasets.

HiCNorm, which defines the potential sources of systematic bias in Hi-C data explicitly, could not correct the biased visibility. Widely used iterative correction method (ICE) scales the contact matrices for equal visibility of each locus, without defining the source of bias at first place [43]. ICE correction could balance the Hi-C signals in condensed and decondensed regions of the contact matrix locally, though at the cost of inadvertent elevation of long-range signals impinging from the condensed chromatin domains. To mitigate this artefact, we suggest a distance-dependent corrective step to be added post ICE-correction. Further, ICE only elevates the obscure signals in the Hi-C matrix uniformly and does not resolve the structured sub-TAD pattern of the condensed domains as observed through GAM. This suggests that computational methods have their own limitations in resolving the obscure signals and Hi-C might need appropriate experimental refinement to resolve the condensed chromatin domains at sub-TAD levels.

Williamson et al. had earlier reported discrepancy between 5C/Hi-C and the DNA FISH results concerning the condensation and decondensation of HoxD locus during embryonic stem cell differentiation. While 5C/Hi-C largely showed that the locus remained condensed, DNA FISH clearly suggested that the locus decondensed upon differentiation [37]. In contrast, Kundu et al. has recently corroborated the 5C and DNA-FISH results on HoxA and HoxD locus. We suggest that the biased efficiency of restriction digestion of condensed and decondensed forms of the locus might underlie these discrepancies. Indeed, we were able to infer the condensed and decondensed states of HoxA and HoxD loci in mESC and NPC respectively from 1D read counts of Hi-C data [29] (Fig. S5h).

While we have shown that the biased visibility in Hi-C datasets can be largely addressed using ICE with an additional corrective step, the uncorrected bias itself is advantageous to explore another layer of chromatin organization. Differential visibility of chromatin domains helps inferring dynamics of condensed and decondensed states of chromatin. Towards this, our analysis on developmental regulation of condensed and decondensed chromatin domains serve as a proof of principle. Coordinated changes in restriction enzyme accessibility and the epigenetic states of the genes contend against the arguments dismissing the observed bias merely a trivial one. We also observed the dynamical changes in the restriction enzyme accessibility during synthetic manipulation of the genome organization. Lamin deletion in the genome is associated with decondensation of LADs [53]. We readily captured the decondensation of LADs in Lamin knock out cells from the 1D Hi-C reads itself.

To identify the sequence features of domain boundaries, we tested the enrichment of several different genomic and epigenomic attributes (Fig. 4b, S5c). We observed the enrichment of CTCF, RAD21, TOP2b, YY1 binding sites and repeat elements like MIR at boundaries, which are well known boundary elements of chromatin domains [11, 14, 58, 59]. These observations collectively reinforce our claim on biological authenticity of condensed and decondensed domains identified outside the LAD regions.

Our proposal that the potential source of the biased visibility is differential restriction digestion can be subjected to criticisms. It can be argued that restriction digestion is generally uniform, but the differential ligation could cause the biased visibility. We do not entirely rule out the possibility that the restriction endonuclease effectively nicks the DNA in condensed domains, but ligase fails to ligate the digested DNA due to stiffness and steric hindrance caused by nucleosomes and heterochromatin proteins in the condensed domains. However, this is not supported by some of our analyses. When we plotted the Hi-C read counts against those of restriction digested chromatin, we observed a linear scaling, while there should have been bias towards the axis of restriction digested chromatin because of presumed efficient digestion and poor ligation in Hi-C experiments. We also propose that the inefficient ligation of digested condensed chromatin could largely be a property of in-situ Hi-C and not the *in-solution* Hi-C, which involves dilution of digested chromatin and condensed chromatin is likely to exhibit relatively loose conformation owing to the usage of detergent and the heat during the dilution step. We, therefore, compared the read counts of in-situ Hi-C and *in-solution* Hi-C datasets obtained from the same study. As shown in the Fig. S3c, we found good correlation between the two, while bias should have been reflected towards the axis representing reads from *in-solution* Hi-C assuming efficient digestion but poor ligation in in-situ HiC. We thus propose that the biased visibility in Hi-C datasets is caused by biased restriction digestion, and not the ligation.

It can also be argued that the cross-linking of proteins with DNA might mask the visibility of restriction sites. To test this hypothesis, we analysed the native Hi-C (in-situ Hi-C without cross-linking step) data from Rao et al. [45]. We observed striking correlation between corrected 1D read-counts of in-situ Hi-C and *native* Hi-C (*ρ* = 0.94 and 0.83 for raw and corrected read counts respectively, Fig. S7a). Accordingly, the distributions of 1D read counts for cLADs and ciLADs exhibited similar patterns in native and in-situ Hi-C (Fig. S7b-c), implying that majority of the observed bias was not merely the consequence of masking of restriction sites by cross-linking. This was further supported by the negative correlation observed between corrected 1D Hi-C read counts and the half-life (t_1/2_) of the chromatin conformations calculated through restriction digestion of chromatin prior to Hi-C in a recently appeared liquid-chromatin Hi-C study (Belaghzal et al 2019, bioRxiv) (Fig. S7d). These observations were also consistent with our observations through data not involving cross-linking and ligation steps, namely RED-seq, which clearly suggested differential digestion of condensed and decondensed domains. We also showed that the biased digestion of the chromatin was not restricted to particular restriction endonuclease. Hi-C datasets generated using 6-cutter (HindIII, NcoI and BglII) and 4-cutter (DpnII and MboI) restriction endonucleases consistently showed the biased visibility (Table S1). Recently Chereji et al. [60] has also shown ~ 30% decrease in the fraction of AluI-digested heterochromatin when compared to active chromatin (fraction cut: 0.24 vs. 0.35 respectively). These observations suggest that the visibility bias pervade to datasets generated through most, if not all, of the widely used restriction endonucleases. Altogether, our observations imply that the bias in 1D read-counts of Hi-C experiments primarily associates with the differential restriction digestion, and not with the masking of restriction sites through formaldehyde cross-linking, the differential ligation efficiency, and the choice of restriction endonuclease.

While the Hi-C data generated through DNase enzyme also exhibit the bias, it is interesting to note that the digestion through Micrococcal Nuclease (MNase) does not exhibit significant bias [61–63] (Fig. S7e). Through analysis of time-course MNase-seq data in drosophila Kc167 cells, we observed significant difference in the corrected read counts of polytene band and inter-band regions during the initial time-points (upto 15 min). This difference diminished gradually and completely disappeared after 40 min of MNase digestion, highlighting successive digestion of compact heterochromatin regions by MNase. We suggest that the smaller size of MNase enzyme (~16KDa as compared to ~30Kda HindIII and DNase I) and its intrinsic specificity to linker region of nucleosomal DNA might implicate in accessing and digesting the heterochromatin in a progressive manner.

## Conclusion

Taken together, the study highlights significant bias in the visibility of condensed and decondensed chromatin domains in Hi-C datasets attributing to non-uniform digestion of chromatin through restriction endonuclease. The existing computational methods fail to correct this bias appropriately and need additional corrective measures. Finally, we show that the repurposing of digestion bias is instrumental in deciphering another layer of gene-regulation through the dynamics of chromatin condensation and decondensation.

## Methods

We did not use any statistical method to predetermine sample size. We did not randomize any experiment. We were not blinded to allocations during experiments and outcome assessment. We mentioned the sample sizes and statistical tests wherever applicable. Source of each dataset is listed in Table S1.

### Processing of RED-seq data

Hi-C includes additional steps like cross-linking and ligation besides restriction digestion. To delineate our claims of biased restriction digestion, we used data generated through sequencing of in-situ restriction digested chromatin and compared with that of *in-solution* restriction digested naked DNA. We obtained the bedgraph files (mm9) of mapped and processed reads of in-situ digested chromatin and *in-solution* digested naked DNA of mESC from GSE51821. The files were converted to mm10 assembly using University of California Santa Cruz (UCSC)‘s ‘liftover’ utility (http://genome.ucsc.edu/cgi-bin/hgLiftOver). The reads were binned into 10 kb bins, which were then corrected for the density of restriction sites and the GC content sequentially in that order, as elaborated in the next section. We also mapped the raw reads (sequence read archive or SRA files) directly to mm10 genome assembly to test if our observations were not the artefacts of lifting over. ‘SRA’ files were first converted to ‘fastq’ files using ‘fastq-dump’ function of ‘SRA toolkit’ (https://www.ncbi.nlm.nih.gov/sra/docs/toolkitsoft/). Reads were mapped to indexed mouse genome (mm10) using bowtie2 (http://bowtie-bio.sourceforge.net/bowtie2/index.shtml). Mapped reads were sorted using ‘sort’ and PCR duplicates were removed using ‘markdup’ functions of SAMtools (https://github.com/samtools/samtools). Mapped reads with quality less than 30 were discarded. The results from bowtie2 mapping were consistent with the results obtained through lifting over the mapped bedgraph data. Since Chen et al. also used additional in-house programs (some of which are now deprecated) to process the RED-seq data, we decided to keep the analysis through lifting over their mm9-mapped and processed data to mm10 in the Fig. 1 in order to maintain the consistency between Chen et al’s and our analyses of RED-seq data. We kept the results of bowtie2-mapped RED-seq data in the supplementary (Fig. S1d).

### Processing of Hi-C datasets and correction of 1D read counts

#### HiCUP processing of hi-C data

Wherever processed Hi-C data was not available, we obtained the SRA files and converted to fastq files using NCBI SRA toolkit. We implemented HiCUP package (http://www.bioinformatics.babraham.ac.uk/projects/hicup/) to process the Hi-C reads. The forward and reverse reads were mapped separately to the indexed reference genome. The data was further filtered for invalid and duplicated read pairs.

#### In-house processing of Hi-C data

To verify if our observations were not specific to HiCUP processing, we also used an in-house pipeline to process the Hi-C reads through bowtie2 (http://bowtie-bio.sourceforge.net/bowtie2/index.shtml). Hi-C paired-end reads were mixed, aligned to the indexed reference genome as single-end reads using bowtie2, and filtered for duplicates using ‘markdup’ function of ‘SAMtools’. Reads with quality score less than 30 were discarded.

#### Calculation and correction of 1D read counts

To calculate 1D read count, we mixed forward and reverse reads and binned into 10 kb bins genome-wide. Since the number of restriction sites (RE-density) in the bins might influence the 1D read counts, we first removed the RE-density associated bias from Hi-C read counts. We used the residuals of read counts after loess regression against RE density of genomic bins. The 1D read counts can also be biased due to varying GC content of genomic bins since GC-rich domains are readily captured in sequencing reactions as compared to GC-poor regions. We removed this bias by calculating residuals of RE-corrected read counts through loess regression against GC content of corresponding genomic bins. The final corrected read count had no scaling against RE density and GC content of the genomic bins as shown in the Fig. S1a-b. We considered the genomic regions with high mappability (≥0.8, 75% of whole genome) for our analysis. The mappability score above this cut-off did not exhibit significant scaling with the corrected 1D read count (Fig. S1c). The corrected read counts followed the Gaussian distribution (Fig. S1e) and we further converted these values to Z-score for plotting purpose.

#### Normalization of atypical systematic biases of Hi-C data

To normalize the known systematic biases in Hi-C contact maps, we used HiCNorm (http://www.people.fas.harvard.edu/~junliu/HiCNorm/) and ‘Iterative Correction and Eigen vector decomposition’ (ICE) packages (https://github.com/mirnylab). Table S1 marks the details of Hi-C datasets and their processing details. To calculate the 1D read count of normalized datasets, we summed the values for each column in the normalized Hi-C contact matrices.

### Analysis of GAM data

We downloaded the normalized contact matrices of 30 kb resolution from GSE64881 and converted to 1D track by summing up the columns of the matrix. We obtained the condensed and decondensed domains inferred from the 1D read counts of mESC Hi-C data of Bonev et al. [33] to make boxplots of contact strengths given by GAM.

### Analysis of MNase-seq data

SRA files were downloaded from GSE128689. The SRA files were converted to fastq files using ‘fastq-dump’ from NCBI SRA toolkit and aligned on to indexed Drosophila genome (dm6) using bowtie2 with ‘very-sensitive’ alignment. The output ‘sam’ files were converted into ‘bam’ files, the reads were sorted using ‘sort’ and duplicates were removed using ‘markdup’ functions of ‘SAMtools’ package. Reads with mapping quality greater than 40 were binned at 5 kb resolution and corrected for GC bias.

### Domain calling

We identified the condensed and decondensed domains using the strategy given by Guelen et al. [64]. In short, read counts were first binarized as + 1 and − 1 depending upon whether the values were positive or negative in the Z-scores. The domain boundaries were identified by subtracting the average of 20 windows on either side of uniformly distributed (per 10 kb) reference points. We determined a cut-off on this value through randomization of the read counts in the genome and keeping the false discovery rate to < 5%. By calculating the relative proportion of positive and negative values in each inter-boundary region, we demarcated condensed and decondensed domains. We set the minimal proportion of either positive or negative values to 0.8 in order to classify the domains as decondensed and condensed respectively.

### Analysis of constitutive LADs and constitutive inter-LADs

We downloaded constitutive LAD (cLAD, *n* = 3843) and constitutive inter-LAD (ciLAD, *n* = 3452) regions of mouse ES cells (mESC), neuronal progenitor cells (NPC) and astrocytes from GSE17051. Similarly, 605 cLADs in human were obtained by comparing LAD coordinates in IMR90 and heterochromatin domain coordinates in h1ESC and K562 cell-lines from ENCODE (https://www.encodeproject.org/comparative/chromatin/). To analyse the alteration in the conformation of cLAD domains, we obtained the WT and the Lamin knockout Hi-C data of mESCs, binned the reads into 10 kb bins and used log ratio of Lamin KO to WT for our analyses.

### Analysis of polytene and normal diploid hi-C data of Drosophila

We downloaded Hi-C SRA files from GSE72510 (polytene, dm6) and GSE63518 (normal diploid Kc167, dm6) and processed using HiCUP pipeline. We binned the reads at 5 kb resolution and corrected for RE-density and GC content as before. We downloaded polytene TADs from Eagen et al. (2015) [50] and lifted over the coordinates to dm6 assembly. We mapped 5 kb bins to polytene TADs and considered those inside TADs as polytene band bins and rest as inter-band bins. We generated raw contact maps at a 5 kb resolution and normalized using HiCNorm and ICE methods as earlier.

### Allele-specific Hi-C analysis of mouse X-chromosome

We downloaded the allele-specific valid DNase Hi-C pairs of brain and patski cells from GSE68992. We removed the reads mapping to both the references and binned the allele-specific reads at 20 kb resolution to obtain one-dimensional read counts. We corrected the read counts for GC content using loess regression as earlier. To visualize the interactions, we generated raw and ICE corrected contact maps at 100 kb. HiCNorm does not suit to DNase-Hi-C data due to the usage of DNase instead of restriction endonuclease, and hence not used.

### Analysis of histone modification and CTCF chromatin immunoprecipitation (ChIP-seq) datasets

Source of ChIP-Seq datasets are given in Table S1. We binned the reads at 10 kb resolution, collated into a table and quantile normalized using *normalize.quantiles* function of *preprocessCore* R-package *(**https://github.com/bmbolstad/preprocessCore**)*. We generated mean enrichment plots by aligning all boundaries that had at-least 200 kb of condensed/decondensed domain on either side. We used R-package *ggplot2 (**https://ggplot2.tidyverse.org/**)* to scatterplot the data.

### Analysis of repeat elements

Repeat elements were obtained from ‘RepeatMasker’ track of UCSC genome browser (https://genome.ucsc.edu/). The mean enrichment, scaled between 0 to 1, of each repeat element was plotted as a function of distance from the boundary of condensed and decondensed domains, at 10kb resolution.

### Distance sorted contact optimization (DiSCO)

We optimized the raw, HiCNorm-corrected and ICE-corrected Hi-C data for the distance dependent bias in the interaction frequencies of condensed and decondensed domains. To balance the interaction frequencies in condensed and decondensed domains, we performed the following steps: i) We first prepared two separate lists of condensed-to-condensed and decondensed-to-decondensed interactions. ii) We prepared the bins of distance spans of the interactions starting from 10 kb to 100 Mb and measured the mean values of pairwise interaction frequencies for each distance bin, individually for each list. iii) The curve of mean interaction frequency as a function of genomic distance served as regression line from which we calculated the residuals for all the pairwise interactions in the two lists. This was achieved by subtracting mean interaction frequency (μ) from the individual interaction frequency (x) at different distance ranges, separately for each list. iv) The mean values for different genomic distances in the two lists were quantile normalized using ‘*normalize.quantiles’* function of ‘*preprocessCore’* R-package (https://github.com/bmbolstad/preprocessCore). v) We finally added the residuals to quantile-normalized mean values to recover the transformed data of individual pairwise interactions in the two lists.

## Supplementary information

**Additional file 1: Table S1.** Details of the datasets used in the study. The universal resource locations (URLs) of NCBI GEO, ENCODE, UCSC genome browser and ArrayExpress are https://www.ncbi.nlm.nih.gov/geo/, https://www.encodeproject.org/, https://genome.ucsc.edu/ and https://www.ebi.ac.uk/arrayexpress/ respectively. **Figure S1.** Related to Fig. 1. (a) Loess correction for the negative scaling of raw read counts against the restriction site (RE) density in 10 Kb genomic bins. Left panel represents data before loess correction and right panel after loess correction of read counts against REdensity (b) Loess correction for the positive scaling of RE-corrected read counts against the GC content of 10 Kb genomic bins. First panel shows scatter plot of GC content vs. REcorrected read counts. Second panel shows scatter plot of GC content vs. GC- and REcorrected read counts. Third panel represents RE-density vs. GC- and RE-corrected read counts. Fourth panel shows scaling of GC- and RE-corrected read counts against the raw read counts. (c) Corrected 1D read count as a function of mappability score (≥0.8). The datasets analysed are mentioned on top of each panel. (d) Analysis of RED-seq data by directly mapping the reads to mm10 assembly of mouse genome. The scatter plot of naked DNA vs. in-situ chromatin re-captures the pattern shown in Fig. 1a. The distributions of corrected read-counts of in-situ digested chromatin and *in-solution* digested naked DNA for cLADs and ciLAD regions echo our observations in Fig. 1b. (e) The distribution of corrected 1D Hi-C read counts in mESC. (f) Size distribution of domains identified through analysis of corrected read counts. Plotted are the mean values with the standard error bars (g) Genomic coverage of condensed domains within constitutive LAD and constitutive inter-LAD regions. Shown are the pie charts of 10Kb bins mapping to cLAD and ciLADs in different datasets. **Figure S2.** Related to Fig. 1. (a) Distributions of raw and corrected read count in cLAD and ciLADs across different in-situ Hi-C datasets in mouse. (b) Distributions of raw and corrected read count in cLAD and ciLADs across different in-situ Hi-C datasets in human. We calculated *p*-values using two-tailed Mann-Whitney U test. **Figure S3.** Related to Fig. 1. (a) Distributions of bowtie-processed raw and corrected read counts in cLAD and ciLADs across in-situ Hi-C datasets of mESC, NPC and CN cells. We calculated p-values using two-tailed Mann-Whitney U test. (b) Side-by-side comparison of raw and corrected read counts mapping to cLAD and ciLADs in in-situ and *in-solution* (dilution) Hi-C datasets obtained for the same cells (mouse fetal liver) from the same study. (c) Scatter plot of corrected read counts obtained from in-situ and *in-solution* Hi-C datasets. **Figure S4.** Related to Fig. 2. (a) Distribution of interaction frequencies of decondensedto-decondensed and condensed-to-condensed interactions as a function of genomic distance in the raw, HiCNorm-corrected and ICE-corrected HiC, and GAM datasets. Upper and lower panels show plots without and with DiSCO corrections respectively. Both axes are log10 tranformed and y-axis was further scaled from 0 to 1 for comparison across plots. (b) Distribution of ICE+DiSCO corrected 1D read counts in the condensed and decondensed domains. (c) Additional examples comparing the corrected 1D read counts and the contact matrices of raw, HiCNorm-corrected, ICE-corrected HiC, and the GAM datasets. (d-e) Additional examples comparing the contact matrices of raw, HiCNorm-corrected, and ICEcorrected Hi-C datasets with and without DiSCO correction. **Figure S5.** Related to Figure4. (a) Scatter plots of corrected read counts in mESC vs. NPC and in NPC vs. CN. (b) Enrichment of histone modifications around boundary between decondensed and condensed domains in mouse cortical neurons (CN). (c) Enrichment of various genomic attributes around domain boundaries and domain centers. (d) Distribution of condensed and decondensed states of chromatin domains during mESC to NPC differentiation. (e-g) Examples of histone modification profiles around ciLAD condensed and decondensed domains in mESC and NPC. (h) Visibility bias at polycomb regulated HoxA and HoxD loci. These loci are condensed in mESC through polycomb proteins, but are decondensed in NPC. The corrected 1D read counts of Hi-C (Fraser et al. 2016) corroborated this pattern. **Figure S6.** Related to Fig. 4. (a) Enrichment of Gene Ontology Process terms among the genes exhibiting condensation (left) and decondensation (right) during ESC-to-NPC transition. Shown are the top 30 terms through ToppGene Suite. Nervous system associated terms are highlighted in brown colour. (b) Significance of overlap between MSigDB gene sets and the genes exhibiting condensation (left) and decondensation (right) during ESC-to-NPC transition. Shown are the top-30 terms through Gene Set Enrichment Analysis (GSEA). Polycomb associated terms are highlighted in brown colour. Vertical dashed line in each plot marks FDR of 0.05. **Figure S7.** (a) Analysis of Native Hi-C data. Scatter plots represents the correlation between 1D reads of in-situ Hi-C and native Hi-C. (b) The boxplots represent the distributions of raw and corrected 1D read counts of in-situ and native Hi-C for the cLAD and ciLAD regions. (c) An example of chromosomal tracks of raw and corrected read counts of in-situ and native Hi-C. (d) Chromosomal tracks of raw and corrected read counts for the region Chr2: 120–240 mb in K562 cell-line. These tracks should be viewed in an approximate alignment to Fig. 4F of *Belaghzai* et al*, bioRxiv 2019.* (e) Distributions of raw and corrected read counts of MNase digested chromatin for polytene band and inter-band regions in drosophila Kc167 cell-line. From left to right are the boxplots for different time points of MNase digestion. It is apparent that after 40 min of MNase digestion, both the band and inter-band regions exhibit similar levels of read counts, implying lack of bias.

## Data Availability

The accession IDs of all the datasets analysed in this study are available in Table S1. The bedgraph files for the corrected read counts of all the Hi-C datasets are available at following link: https://bitbucket.org/ken_at_keerthivasan/compaction_from_hic/downloads/ The computer programs used in the analysis are available at: https://github.com/rckeerthivasan/compaction

## Abbreviations

1D: One-dimensional
3C: Chromosome Conformation Capture
3D: Three-dimensional
4C: Circular Chromosome Conformation Capture
5C: Chromosome Conformation Capture Carbon Copy
ChIP: Chromatin Immuno Precipitation
ciLAD: constitutive inter Lamina Associated Domains
cLAD: constitutive Lamina Associated Domains
CN: Cortical Neurons
DiSCO: Distance Sorted Contact Optimization
GAM: Genome Architecture Mapping
H3K27me3: Histone-3-lysine-27-tri-methylation
H3K9me3: Histone-3-lysine-9-tri-methylation
Hi-C: High-throughput Chromosome Conformation Capture
HiCUP: Hi-C User Pipeline
ICE: Iterative Correction and Eigen Vector decomposition
KO: Knock Out
mESC: mouse Embryonic Stem Cell
MIR: Mammalian-wide Interspersed repeats
NPC: Neuronal Progenitor Cell
RE-density: Restriction Endonuclease site density
RED-seq: Restriction Endonuclease Digestion coupled with sequencing
SRA: Sequence Read Archive
TAD: Topologically Associated Domain
UCSC: University of California Santa Cruz
WT: Wild-Type
